# Actively Induced Supercoiling Can Slow Down Plasmid Solutions by Trapping the Threading Entanglements

**DOI:** 10.1021/acsnano.5c10811

**Published:** 2026-05-15

**Authors:** Roman Staňo, Renáta Rusková, Dušan Račko, Jan Smrek

**Affiliations:** † Faculty of Physics, 2152 University of Vienna, Boltzmanngasse 5, 1090 Vienna, Austria; ‡ Yusuf Hamied Department of Chemistry, 87171 University of Cambridge, Lensfield Road, Cambridge CB2 1EW, U.K.; § Polymer Institute, Slovak Academy of Sciences, Dúbravská cesta 9, 845 41 Bratislava, Slovakia; ∥ Department of Physics, 27258 Sapienza University of Rome, Piazzale Aldo Moro 5, 00185 Rome, Italy

**Keywords:** DNA, topology, ring polymer, supercoiling, gyrase, active matter, glass

## Abstract

Harnessing the topology of ring polymers as a design motif in functional nanomaterials is becoming a promising direction in the field of soft matter. For example, the ring topology of DNA plasmids prevents the relaxation of excess twist introduced to the polymer, instead yielding helical supercoiled structures. In equilibrium semidilute solutions, tightly supercoiled rings relax faster than their torsionally relaxed counterparts, since the looser conformations of the latter allow for rings to thread through each other and entrain through entanglements. Here we use molecular simulations to explore a nonequilibrium scenario, in which a supercoiling agent, akin to gyrase enzymes, rapidly induces supercoiling in the suspensions of relaxed plasmids. The activity of the agent not only alters the conformational topology from open to branched, but also locks in threaded rings into supramolecular clusters, which relax very slowly. Ultimately, our work shows how the polymer topology under nonequilibrium conditions can be leveraged to tune dynamic behavior of macromolecular systems, suggesting a method to create a class of driven materials vitrified by activity.

## Graphical abstract

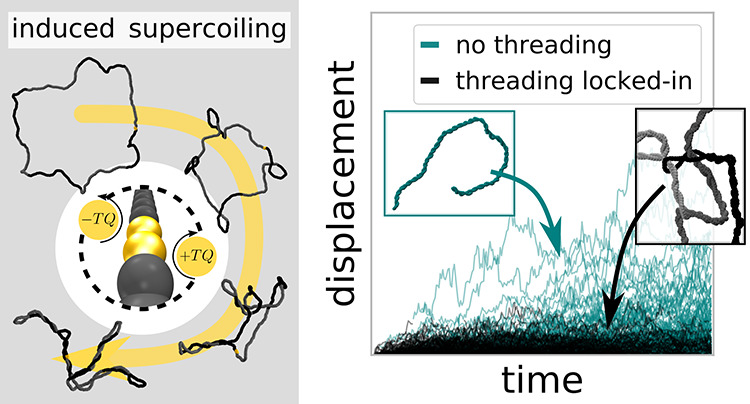

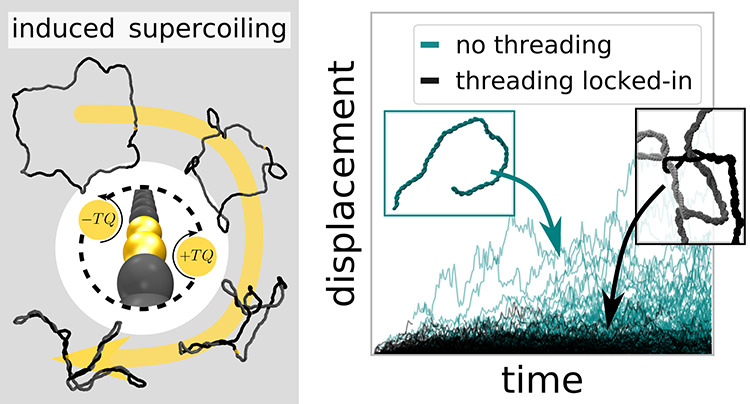

## I Introduction

Understanding the topological constraints and entanglements in polymer solutions and melts under diverse conditions is among the biggest open problems in the field of soft matter.^1–4^ Different forms of entanglement and entrainment can enhance or diminish certain relaxation modes, in turn affecting the viscoelastic properties of polymer materials,^5–13^ but also the dynamics of other classes of fibrous matter such as weaved molecular sheets,^14^ macroscopic worms,^15–17^ ropes or soft robots.^18,19^ The ability to select the relaxation pathways would allow to tune the properties of the solution without the need to change the polymer chemistry. The entanglements and the ensuing dynamics are many-body in nature and crucially depend on the chain topology^20^ and external^21^ or internal driving.^22^ These reasons make a theoretical prediction of the properties challenging, but concomitantly present vast possibilities for diverse modulation of the behavior.

In particular, polymers of *ring* topology that are unknotted and mutually nonconcatenated have been studied extensively as a prototypical example.^1,23^ The well-studied equilibrium relaxation of linear polymers (reptation) proceeds by one-dimensional diffusion along the chains contour, while the transverse motions are restricted by the entanglements with the other chains. While rings can also exhibit linear-like entanglements,^24^ the rings have no ends, hence the reptation mechanism is altered.^25–28^ Additionally, rings allow for *threading* entanglements when one chain enters the opening of another one, but the exact contribution of threadings to the viscoelasticity is not fully understood. To satisfy the constraints of mutual ring nonconcatenation, at semidilute concentrations the rings adopt branched configurations^29,30^ that are however not tightly double-folded and include mutual threadings.^31^ On the one hand, treating rings in melts as branched annealed trees without threading,^9,32^ or other models that neglect threadings,^26^ reproduce the experimental moduli almost quantitatively, suggesting that threading relaxation does not contribute significantly to viscoelastic properties. On the other hand, the theories without threadings predict faster ring diffusion^25–27,32^ than observed^33^ and molecular simulations^30,34–37^ show that threading is present in polymer melts, and the threaded rings do diffuse slower than their unthreaded counterparts. Recent studies show that stiffer rings can exhibit prolonged subdiffusive regime and glassy dynamical features.^37–41^ The latter is strongly enhanced also in certain ring systems out of equilibrium, either driven externally by extensional flows^20,42^ or internally, for example in active topological glass of block copolymers with hot and cold blocks.^43,44^ In both of the above nonequilibrium systems, the dynamics effects have been directly linked to threadings, as the flow field or activity can turn threadings into deadlocks^44^ – geometrically entangled constructs with very long relaxation times, causing either large increase in viscosity or even vitrification of the whole system. Hence active induction of threadings can slow down the system and impact the viscoelasticity, but is difficult to reverse or regulate to achieve a control.

A promising candidate for such a regulation is the supercoiling^45–47^ that can be present in ring polymers with both bending and torsional elasticity, readily realized with circular DNA plasmids or other ribbon-like polymers. In such *equilibrium* solution, the abundance of linear-like and threading entanglements can be controlled by the degree of supercoiling. The enhanced supercoiling decreases the threadable area of the rings and stiffens their effective backbone, leading to a decrease of both, the threadings and the entanglements, and thereby speeds up the system.^48^ The supercoiling can be also induced after the polymer synthesis providing the sought-for control over the properties. In the case of DNA, this can be achieved by molecular motors such as gyrase^49,50^ or RNA polymerase,^51^ or intercalators.^52–55^ The detailed action of these supercoiling agents can be different. For example, while the intercalator is inserted in the base pairs and thereby changing the bending-torsion balance, gyrase temporarily cuts the DNA strand, repositions the chain under- or overcrossings, and then binds the cut parts together, thereby inducing excess torsion.^56^ For other ribbon-like polymers the supercoiling can be induced by the incorporation of artificial molecular motors in the polymers backbone.^57,58^ While the equilibrium consequences of the supercoiling are known, the impact of the active induction of supercoiling on the threadings and dynamic properties of such ribbon-like ring solutions remains unexplored. We do not aim at modeling a specific supercoiling agent, but rather focus on the universal properties that arise from the coarse-grained perspective of the supercoiling induction.

The crucial parameter in considering the role of the active process is the time to reach the supercoiling steady state, when the active forces are balanced by the interactions, in comparison to the polymer relaxation time. Taking gyrase as an example of the supercoiling agent, it operates on the scale of seconds^59–61^ providing enough time for short plasmids (<100 kbp) to equilibrate on the scale of the diffusion time in aqueous solution as measured in diffusion experiments.^62–64^ Yet, the gyrase operation rate is energy- and tension-dependent, and for longer plasmids or solvents of higher viscosity the relaxation times can be orders of magnitude longer. Under these conditions, the supercoiling process operates far from equilibrium, potentially leading to diverse dynamical properties. To understand the impact of active induction of supercoiling and to explore its potential use for the control of polymer solution dynamics, we focus on this regime where the supercoiling is induced faster than the diffusional relaxation. We investigate a solution of ring polymers actively driven by external torques applying excess twist to the contour and we assess its impact on the conformations, dynamics and the entanglement properties. We show that depending on the active torque, the conformational changes from open rings to branched and linear-like supercoils can bring about metastable states affecting the dynamics in a fashion similar to arrest of topological glasses.^37,43^ The plasmid system we focus on is not only biologically interesting but also falls within a broader field of prospective DNA nanotechnology augmented by enzyme activity or external fields.^5,10,65–67^

## II Results and Discussion

We represent the ring polymers using bead–spring models with bending and torsional stiffnesses. We use the models,^68,69^ detailed in (Section 4.1) and SI (Section I B) where we also show that the fine model details affecting microscopic dynamics keep the large-scale properties and phenomena we report here largely unaffected. The models are characterized by torsion between consecutive pairs of monomeric units modeled using a dihedral potential, controlling the preferred pitch along the chain *p* = 2π/ψ_0_, where ψ_0_ is the angle for which the dihedral potential has the minimum. In equilibrium, by choosing appropriate ψ_0_, we can control the supercoiling density σ, which is a measure of the total torsional-bending stress contained within the polymer, defined as σ = 1/*p* = |Lk|/*N*, where Lk is the (excess) linking number Lk = *N*/*p*.^48^ We first simulate 200 equilibrium flat ring ribbons (ψ_0_ = 0) of length *N* = 400 in solution with monomer density ρ *s* ^3^ = 0.08 (Figure 1a), corresponding to the overlap parameter ρ *R*_g_^3^/*N* of about 4.4, signifying semidilute regime with threading entanglements.^48^ Subsequently, we apply the activity on each ring through a supercoiling agent, such as gyrase. Our simulations do not follow the exact microscopic pathway of this enzymatic process^56^ that involves double strand passage and occurs in bursts with waiting times due to structural changes of the motor.^59^ As mentioned, we rather explore its large-scale and coarse-grained consequence, being the change of the supercoiling density σ. Such models with active swivels introducing supercoiling by gyrase or transcription were used previously to address biological problems like unknotting,^70^ formation of topologically associating domains^71^ and cohesin loop extrusion.^72^ To apply the motor enzyme, we select a pair of consecutive monomeric units, we remove the dihedral potentials constraining the pitch of this pair, and we apply oppositely oriented torques on the monomers (Figure 1b and Section 4.2), rotating the monomers around the bond vector. The accumulated angle ψ_*a*_, defines the pitch *p* = 2π/(ψ_a_/*N*) and supercoiling density σ = 1/*p* in the nonequilibrium situation. The activity of the motor is controlled by the value of the fixed applied torque *TQ* (see Section 4.2).

**Figure 1:**
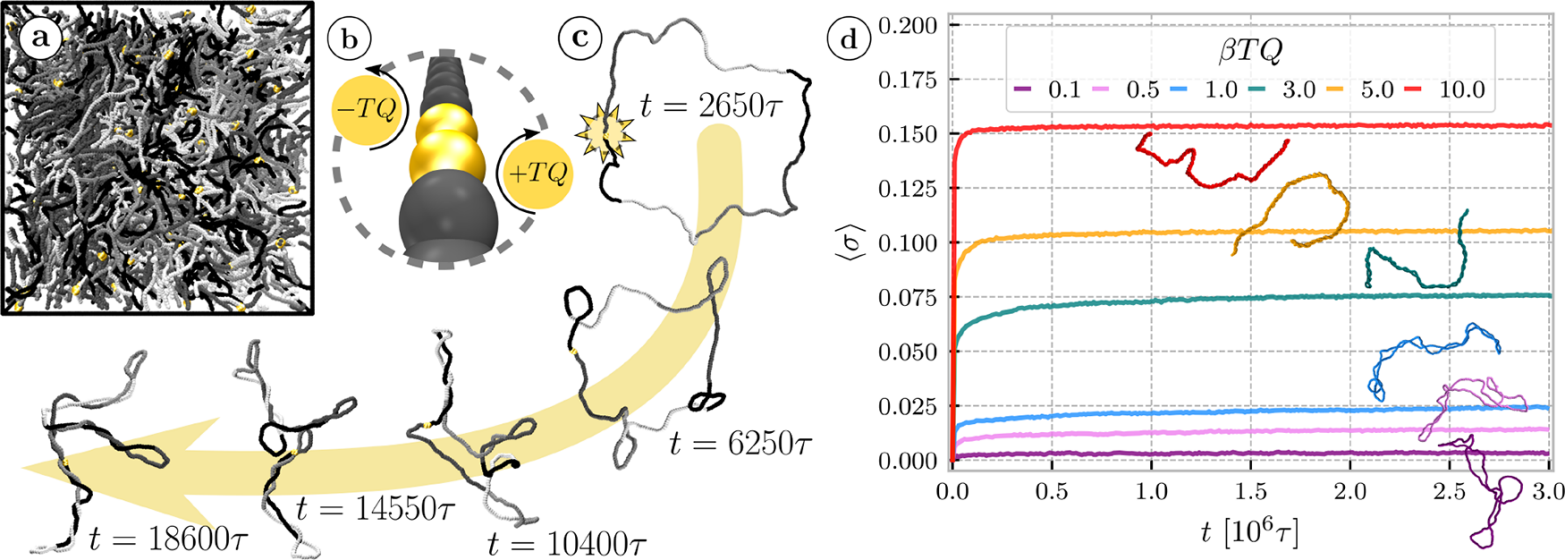
(a) Snapshot of the equilibrium system with fixed σ = 0 with different rings in shades of gray and gold beads representing the pair of monomers where the active torque is subsequently applied. (b) Scheme depicting the application of the active torque (c**)** Time evolution of conformations of a single ring of *N* = 400 in infinite dilution with β*TQ* = 3 applied. (d) Mean supercoiling density σ as a function of time for different values of active torque *TQ*. Snapshots show typical polymer conformations in the dense systems at 2 × 10^6^τ at different torques.

At low *TQ* the activity is not strong enough to significantly overcome the thermal fluctuations and the supercoiling density σ fluctuates around zero, since the ribbon has zero excess supercoiling (ψ_0_ = 0). At higher *TQ* the supercoiling initially grows in time (Figure 1c,d) until it reaches a steady state at which the applied torques are balanced by the bending and torsional stiffness, not allowing further supercoiling. Eventually, for sufficiently large *TQ*, we reach values |σ| ≈ 6% (or even larger) corresponding to the maximal supercoiling density commonly observed in DNA,^56^ with typical plectonemic structures with a high writhe resembling double folded chains or trees with small number of branches (see snapshots in Figure 1d). The highest torques and the resulting highest induced supercoilings we find are on the limit of structural stability of the DNA,^73^ but our results apply to other ribbon-like polymers beyond DNA. The driven transition from the flat ribbon to the supercoiled one is rather fast and, even at low values of *TQ*, takes less than the self-diffusion time in dilute conditions τ_*R*_ = *R*_*g*_^2^/*D* ≃ 6 × 10^4^τ, which we measured in independent simulations.

In equilibrium semidilute solutions, the flat ribbon rings (σ = 0) are rather swollen and of elongated prolate shape (Figures 1 and S3) with large opening and numerous threadings and interpenetration. The equilibrium dynamics, quantified by center-of-mass mean-squared displacement *g*_3_(*t*)­

1 $$g_{3}\left(t\right)=\left\langle {\left[r_{C}\left(t\right)-r_{C}\left(0\right)\right]}^{2}\right\rangle$$

is subdiffusive for short times and diffusive one for longer times, with the crossover time τ_*D*_ ≃ 10^6^τ (Figure 2a). In comparison to ref 48, our present estimate of τ_*D*_ is less impacted by finite system size effects, as our systems are factor of 4 larger.

**Figure 2:**
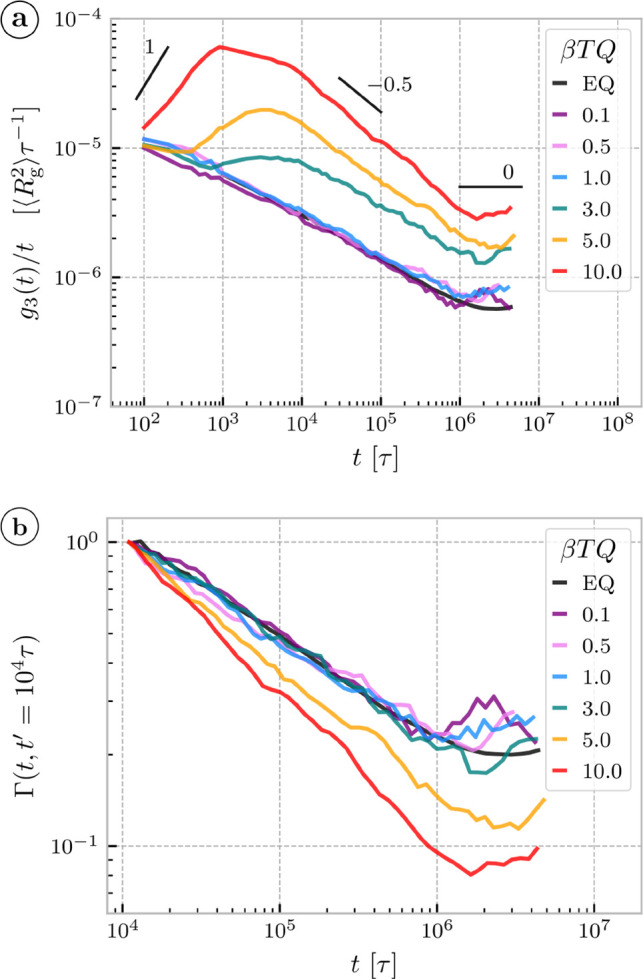
(a) Mean square displacement (eq 1) divided by time since the onset of the activity in the units of mean square radius of gyration over monomer relaxation time for different torques. EQ stands for the equilibrium system with no driving. (b) Relaxation functions (eq 2) for *t*′ = 10^4^τ, probing the dynamics after saturation of supercoiling and after the sharp initial drop in threadings. The equilibrium system has rings in flat ribbon configuration (σ = 0). The data of the equilibrium system are also time-averaged and active systems with β*TQ* ≤ 1 are averaged over four system replicas with different initial conditions.

In contrast to equilibrium, we find diverse dynamical behavior for actively supercoiled rings depending on the value of the active torque, as measured by *g*_3_(*t*) from the point of activity onset, without time averaging for the active systems (Figure 2). Systems with low active torques β*TQ* ≤ 1.0 are essentially identical to the equilibrium system with σ = 0. The seemingly larger fluctuations at late times in comparison to equilibrium are caused by the time-averaging that we applied to the equilibrium system only. Systems with high active torques β*TQ* > 1 exhibit early superdiffusive behavior that crosses over to distinct subdiffusive regimes.

The short-time behavior (*t* < 10^5^τ) is readily explained by the induced level of supercoiling (Figure 1d). In equilibrium, the conformations are weakly doubly folded and branched due to the constraint that each ring must remain nonconcatenated with the others.^31^ High active torques induce strong supercoiling that propagates along the chain and creates ring-scale rearrangements from weakly to tightly doubly folded conformations (Figure 1d). Additionally, the enhanced intrachain interactions induce rapid stiffening of the effective chain^48^ that although does not manifest itself strongly in the overall size *R*_g_, but does so in the shape anisotropy of the conformation (Figure S3). In contrast, low torque systems are only weakly supercoiled and, being already partly doubly folded, their shape transformation is less significant.

In the long-time regime (*t* > 10^5^τ) the supercoiling degree almost already reaches the steady state but the overall dynamics contrasts with that of the equilibrium simulations. In equilibrium melts highly supercoiled plasmids diffuse *faster* than their less supercoiled counterparts.^48^ Here we find that the active melts exhibit a torque-dependent subdiffusive regimes. We observe (Figure 2b) the rings with higher active torque (β*TQ* > 3) show stronger slowdown as measured by the relaxation function Γ­(*t*, *t*′)

2 $$\Gamma \left(t,t^{\prime }\right)=\frac{g_{3}\left(t\right)}{t}\cdot \frac{t^{\prime }}{g_{3}\left(t^{\prime }\right)}$$

a normalized version of the *g*_3_(*t*) /*t* to the value at time *t*′ = 10^4^τ corresponding to the end of the regime of large-scale structural relocations. The stronger relaxation slowdown for the rings with high torques (supercoiling) is in sharp contrast to the relaxation process of equilibrium rings, with corresponding matching σ, exhibiting *speedup* for rings of higher supercoiling (see the equilibrium relaxation function in Figure S12).

To explain the contrast we investigate the interchain threading constraints by means of evaluating intersections of a ring’s contour with another ring’s disk-like minimal surface (see Section 4.4 and ref 31). In Figure 3a, the mean number of rings threading a ring as a function of time exhibits two regimes. The initial (*t* < 10^5^τ) sharp drop coincides with the fast structural rearrangements after the activity onset and, as expected, affects mostly shallow threadings that vanish in this regime (Figure S8). The subsequent slow threading relaxation indicates that larger displacements are necessary to release the remaining threadings but such rearrangements are likely restricted by the threadings themselves. To explore such collective constraints we categorize rings into threading clusters, if ring A threads ring B they belong to a common cluster. As expected from the large number of threadings per ring, the systems at β*TQ* ≤ 1.0 form a fully connected network – all rings belong to the largest cluster at all times Figure 3b. A percolating threading cluster would indicate a possible vitrification or gel-like response if the threadings were hindering the rings motion significantly as found in refs 37,43,44,74 for different systems, but not here at low torques. At higher active torques, where the threadings are tight due to supercoiling and restrict the ring motion significantly, we expected that a high fraction of rings belonging to the largest cluster, *f*_max._ would be predictive of the system slowdown. Contrary to these expectations, the initially fully connected system disintegrates into individual dangling rings and smaller clusters. Moreover, the higher the torque, the *sooner* the largest cluster disintegrates (Figure 3b) but, surprisingly, the stronger the subdiffusive slowdown is (Figure 2b). We checked that the cluster structure and the order of the disintegration, remains qualitatively the same even if the criterion of a cluster is based on threadings of a certain critical depth (Figure S9). Nevertheless, we do find that the membership to a cluster does impact the ring dynamics, even at later stages when the clusters are certainly not percolating. To prove that we plot the *g*_3_(*t*) for each ring separately and at a given time *t* we compute separately the mean over rings in clusters and the mean over dangling rings. As shown in Figure 4a (also Figures S10 and S11 for all torques) the resulting *g*_3_(*t*) of the rings from threading clusters is consistently below the total *g*_3_(*t*) and the dangling rings constitute the more mobile component. Moreover, not only is the *g*_3_(*t*) of the rings in clusters lower, it also grows in time more slowly than the *g*_3_(*t*) of the free counterpart. Comparison across different torques (Figure S11) shows that both, the dangling and the threaded classes are slower for higher torques.

**Figure 3:**
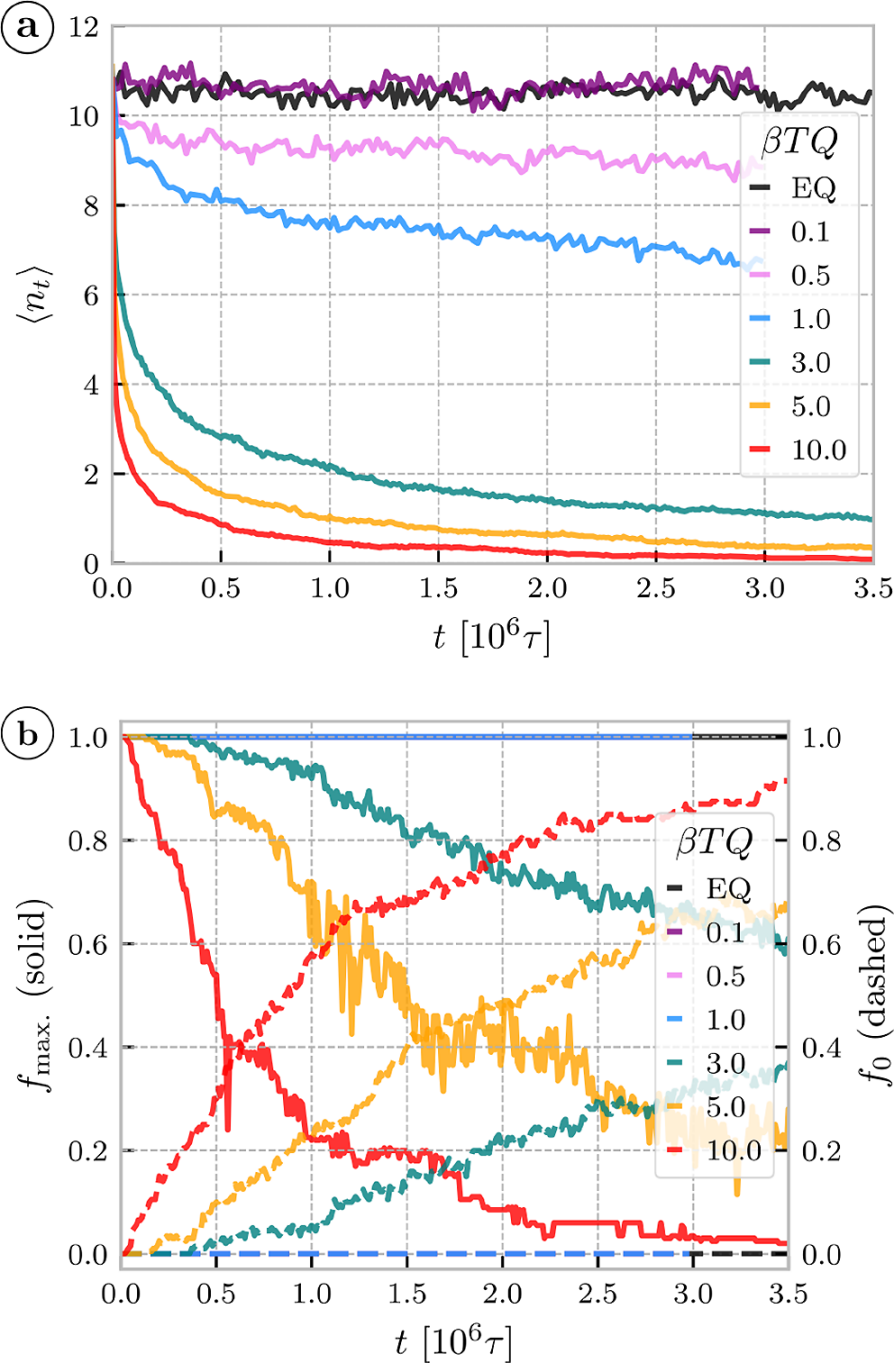
(a) The mean number of threadings per ring as a function of time after the onset of the activity, shown for different torques. EQ stands for the equilibrium system with no driving. (b) Fraction *f*_max._ of the rings belonging to the largest continuous cluster of threadings present in the system (solid, left axis) as a function of time. Fraction *f*_0_ of dangling rings (dashed, right axis), rings with no threading, as a function of time. In both cases, the criterion for threading is nonzero threading depth (separation length), defined by eq 11, *L*_sep_^thr^, the effect of this threshold is explored in the SI (Figure S9).

**Figure 4:**
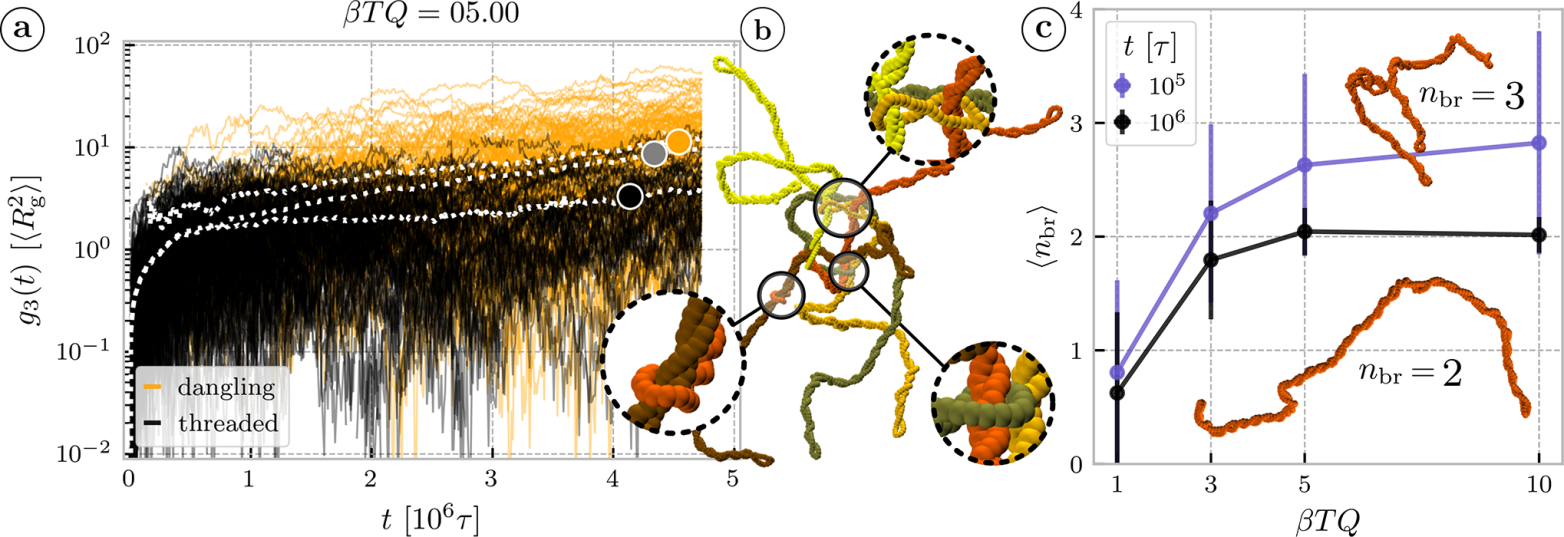
(a) The mean square displacement (eq 1) of all individual rings, with the point at time *t* colored black if the ring is threaded or orange if it is dangling. Three white dotted lines show means over three subpopulations of rings–over all dangling rings, over all threaded rings and over all of the rings, marked with orange, black and gray circular marker, respectively. (b) Snapshot showing long-lived and deep locked-in threadings, magnified in the insets, in a cluster of rings (different colors) at 2 × 10^6^ τ for β*TQ* = 5. (c) Mean number of branches per polymer as a function of torque for two different times–after establishing the steady state of supercoiling and during the stage of slow aging. The error bars are the standard deviations over the ensemble of ring conformations. The inset snapshots show a conformation of a selected ring at β*TQ* = 5 in the time *t* = 10^5^τ where it has three branches and the same ring at *t* = 10^6^τ where it relaxes to a doubly folded, linear-like chain.

While clearly the rings in threading clusters are slower, why are then the systems with higher torques and hence more dangling rings slower? We identified two distinct mechanisms underlying this slowdown, distinguished by whether the effect originates from the topology of the threading constraint network or from the topology of the chain itself. First, the threading cluster network topology can constrain the unthreading process to follow a sequential pathway, with this effect becoming more pronounced at higher torques where the network topology is more restrictive. The snapshot in Figure 4b illustrates such a cluster, which due to mutual threading constraints can disassemble only sequentially (brown chain first). Second, in contrast to the network-based constraints, the chain topology can limit the relaxation. As active torque increases, the resulting higher supercoiling not only tightens the threadings, thereby increasing effective friction, but more importantly can induce geometrical/topological changes of the conformation, constraining the available relaxation modes.

To investigate the impact of the threading network topology on the dynamics we considered an adapted version of the effective 1D topological glass model proposed by Lo and Turner.^75^ There, each ring is represented by a doubly folded chain of linear-like topology, an accurate description of highly supercoiled equilibrium chains of this length.^48^ Each chain can move along its contour and thread or be threaded by another chain. The latter, passive threading, at the end of a chain, such as the threading of orange by the brown chain in Figure 4b, prohibits the curvilinear motion of the threaded chain and hence its disengagement from the cluster. We constructed the corresponding Monte Carlo (MC) simulation (detailed in Section 4.5), where in a single step each chain attempts to move in a curvilinear direction and the move is rejected if the chain contains such a passive threading. If the threading is not at the end of the chain, the move is accepted and the location of the threading is updated in the direction corresponding to the move. If actively threading chain moves out of the threaded chain, the mutual threading vanishes. In contrast to ref 75 that was focusing on equilibrium, we do not allow the creation of new threadings, as these are highly unlikely for the present highly supercoiled case,^48^ and we track only the relaxation from the given threading network conformation that we have from our Molecular Dynamics simulations. Our MC simulations show that the threading networks are potentially vitrifying the system, but again, with trends contrasting with our observations. The effective MC model predicts slower dynamics for the lower torques (SI section I A), consistent with the Lo and Turner model where the relaxation time grows with the number of threadings, the latter being indeed higher for lower torques in our MD systems (Figure 3a).

We find that the Lo and Turner model is not applicable in our case, because of the induced *internal* geometrical and topological changes of the conformations. The 1D contour diffusional relaxation (reptation) modes would be restricted in case of a branched conformations because the branches would have to “squeeze” through the threading openings in order to reach the chain end. We therefore measured, using local writhe (Section 4.4), the topology of the supercoiled chains, characterized by the number of branches *n*_br_, at the onset of the slow relaxation regime (*t* = 10^5^τ). We find that not only the mean number of branches grows with the torque β*TQ*, but the width of their distribution does so too due to highly branched outliers (Figures 4c and S5 for the distribution details). In contrast, at late stages (*t* = 10^6^τ) when many of the initial threadings are relaxed (Figure 3a), the conformations are mostly linear-like, consistent with the equilibrium observations.^48^ Similarly, the system with β*TQ* = 3, separating the behavior of low and high-torque systems, confirms that number of branches close to two already at early times 10^5^τ, translates into equilibrium-like dynamics later (see also Figure S6 for finer time resolution of branching statistics, separately for threaded and dangling rings, and its effect on the individual ring dynamics).

These results together show that the stronger active torques couple the threading and branching features, characterizing the inter- and intrachain topologies of the supercoiled systems. Not only are some of the pre-existing threadings locked-in, but their subsequent relaxation is hindered by the induced branching causing a slowdown of the global system dynamics.

## III Conclusions

Our investigated systems induce supercoiling much faster than the relaxation time resulting in nonequilibrium conformations and partial locking of threadings. Whether such effects survive for slower supercoiling dynamics is not clear at the moment, despite the observation that highly supercoiled rings have few threadings in equilibrium,^48^ because the threading nature and the resulting threading constraint network can be complex functions of the supercoiling rate. Moreover, going beyond a simple constant rate or torque, and inducing the supercoiling with a time-dependent protocol, can have a different impact on the local threading and branching statistics, indicating a possible way to tune the global dynamics by the nonequilibrium driving. The present work is hence the first step in this direction.

In the limit of long rings and high densities we conjecture even stronger slowdown than the one observed here for moderately long rings. Although the rings in a melt adopt compact conformations,^76^ meaning a saturating number of (threading) neighbors in the asymptotic limit, the threading depth statistics is a power-law indicating the existence of many deep threadings.^30,31^ If these threadings are locked-in by strong induced supercoiling they might possibly lead to a complete vitrification of the system. Moreover, highly supercoiled conformations of long rings are branched for entropic reasons^77^ and the branch retraction and contour diffusion are slow processes themselves.^78^ The question however remains if sufficiently many threadings with branched-enough conformations on either side of the threaded chain can be trapped. While there are possibly many ways the threading or branching network can be manipulated, such as use of different stiffness or multiple supercoiling agents (e.g., enzymes) on a single ring, the future theoretical crux of the problem is to understand the connection of the branching and threading statistics with their dynamical consequences.

Some works on equilibrium ring melts indicate that the slow and glassy dynamics emerging from threadings is a nontrivial function of the rings stiffness and concentration.^38–40^ For the same reason, it would be interesting to investigate also blends of rings with different stiffness, fraction of active rings and higher concentrations. The concentration, in particular, can be a crucial parameter in tuning the behavior. While below the overlap concentration, there can be hardly any threadings locked-in and hence no slowdown, one can expect a stronger slowdown as a function of overlap, as the number of threadings is expected to be proportional to the overlap parameter. Yet, higher concentrations at the same chain stiffness might induce nematic ordering of the supercoiled conformations^48^ and hence the behavior can be more complex.

Older works^33^ indicate that even in melts of semiflexible rings exist ring pairs that remain spatially proximal for times significantly exceeding τ_*D*_. Recent simulations^79^ link this observation to ring pairs with geometrically deadlocked threading conformations that relax on time scales surpassing the diffusion time. Inducing supercoiling in these pairs would likely prolong the relaxation even more. Our present work shows how the actively induced supercoiling controls the dynamic response of the polymeric material and it indicates that quickly supercoiled long rings might be a path to create an active topological glass in experiment. Whether it could be achieved also with a gyrase operating at longer time scales or with other faster supercoiling agents remains currently unknown.

## IV Methods

### IV.I Microscopic Model of Plasmid Suspension

We model the DNA plasmids using the standard bead–spring coarse-grained model of a polymer^80,81^ with elements of elastic twistable ribbon.^48,68^ A single plasmid consists of *N* = 400 connected monomeric units, forming an unknotted ring polymer. Each pair of monomeric units at instantaneous distance *r* interacts with an isotropic pair (WCA) potential

3 $$U_{\mathrm{WCA}}\left(r\right)=4\varepsilon \left[{\left(\frac{s}{r}\right)}^{12}-{\left(\frac{s}{r}\right)}^{6}+\frac{1}{4}\right]\cdot H\left(2^{1/6}-\frac{r}{s}\right)$$

where *H*(·) is the Heavside step-function, ε = *k*_B_T = 1/β = 1 sets the energy scale and *s* = 2.5 nm ≈ 7.4 bp sets the length scale of the model, yielding ≈3.0 kbp for the whole plasmid. The bonds between monomeric units are emulated using finitely extensible nonelastic (FENE) bonds

4 $$U_{\mathrm{FENE}}\left(r\right)=-\frac{1}{2}K_{\mathrm{FENE}}R_{0}^{2}\ln\left[1-{\left(\frac{r}{R_{0}}\right)}^{2}\right]$$

where *K*_FENE_ = 40*k*_B_ *T*/*s* ^2^ and *R*_0_ = 1.6*s* is the maximal allowed bond length beyond which the potential diverges. To simulate the polymer contour bending, every triplet of consecutive monomeric units interacts with Kratky–Porod angular potential

5 $$U_{\mathrm{bend}}\left(r\right)=K_{\mathrm{bend}}\left(1-\frac{t_{i}\cdot t_{i+1}}{\left|t_{i}\right|\left|t_{i+1}\right|}\right)$$

where *K*_bend_ = 20 *k*_B_ *T* and the bond vector **t**_*i*_ = **r**_i+1_ – **r**_*i*_, where **r**_*i*_ is the position of the monomeric unit 0 ≤ *i* < *N*, and further we also assume 0 ≡ *N* to simplify the notation in summations running over the whole ring. The persistence length resulting from the above potentials is *l*_p_ ≈ 20*s* ≈ 50 nm ≈ 150 bp.

To embed the torsional elasticity into the DNA chain, each monomeric unit is inscribed three vectors [**u**, **f**, **v**] forming a right-handed orthonormal set of axes,^68^ giving the orientation to the otherwise spherically symmetric particle as shown in Figure 5. For each pair of consecutive monomeric units, we apply a stiff tilting potential

6 $$U_{\mathrm{tilt}}=K_{\mathrm{tilt}}\left(1-u_{i}\cdot \frac{\left(u_{i}-t_{i}\right)}{\left|t_{i}\right|}\right)$$

with *K*_tilt_ = 200*k*_B_ *T* locally aligning **u**_i_ with the bond vector **t**_i_ of the pair as shown in Figure 5. Finally, we apply two dihedral potentials on each pair of consecutive monomeric units

7 $$U_{\mathrm{torsion}}=K_{\mathrm{torsion}}\left(1-\frac{\left(t_{i}\times f_{i}\right)\cdot \left(t_{i}\times f_{i+1}\right)}{\left|t_{i}\times f_{i}\right|\left|t_{i}\times f_{i+1}\right|}\right)$$

8 $$U_{\mathrm{torsion}}=K_{\mathrm{torsion}}\left(1-\frac{\left(t_{i}\times v_{i}\right)\cdot \left(t_{i}\times v_{i+1}\right)}{\left|t_{i}\times v_{i}\right|\left|t_{i}\times v_{i+1}\right|}\right)$$

with *K*_torsion_ = 50*k*_B_ *T* giving the chain the torsional stiffness as shown in Figure 5. Since the monomeric units have internal orientation, they also possess the rotational degrees of freedom controlled by the angular forces originating from the tilting and dihedral potentials. The supercoiling density within the DNA chain is defined as σ = Lk/*N*, where Lk is the linking number^82,83^ calculated as

9 $$\mathrm{Lk}=\frac{1}{4\pi }\oint_{C_{1}}\oint_{C_{2}}\frac{r_{1}-r_{2}}{|r_{1}-r_{2}{|}^{3}}\left({ṙ}_{1}\times {ṙ}_{2}\right)ds_{1}ds_{2}$$

where **r**_*i*_ = **r**_*i*_(*s*_*i*_) is a parametrization of curve *C*_*i*_ with arc length *s*_*i*_ and **ṙ**_*i*_ = **ṙ**_*i*_(*s*_*i*_) = d**r**_*i*_(*s*_*i*_)/d*s*_*i*_.^1^ The curve *C*_1_: **r**_1_(*s*_1_) follows the polymer contour and *C*_2_: **r**_2_(*s*_1_) = **r**_1_(*s*_1_) + ϵ**u**_1_(*s*_1_) where ϵ ≪ 1.

**Figure 5:**
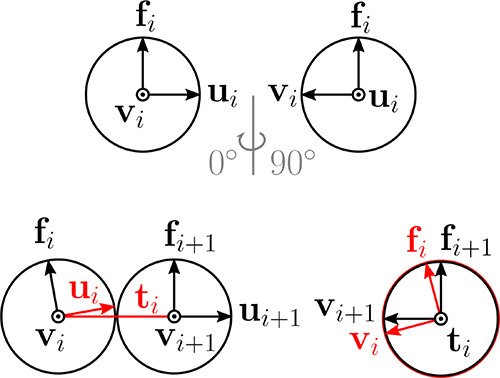
Top: monomeric unit with the set of orientation vectors, bottom left: scheme showing the vectors involved in the tilting potential (eq 6), bottom right: scheme showing the vectors involved in the dihedral potential (eqs 7 and 8).

The solution of M = 200 plasmids is enclosed in a cubical box with periodic boundary conditions and with dimension *L* = 100 *s* ≈ 250 nm, resulting in the monomer concentration ρ*s* ^3^ = 0.08 corresponding to ρ/ρ* ≈ 4.4 in terms of polymer concentration relative to the overlap one, herein defined as ρ* = *N*/*R*_g_^3^ with *R*_g_ being the radius of gyration of a ring with σ = 0, or corresponding to weight concentration of *c*_m_ ≈ 40 mg/mL of DNA.

### IV.II Supercoiling Agent Activity

To simulate the nonequilibrium effect of the supercoiling agent, for each of the plasmids, we select a pair of consecutive monomeric units and remove the dihedral potentials (eqs 7 and 8) from this pair. Simultaneously, we apply an external torque *TQ* on the two above monomers, rotating one of them clockwise and the other one counterclockwise along the bond vector between them. The forces stemming from the applied torque are applied at the distance 0.80σ from the center of the particle, which considering *s* ≈ 2.5 nm and *T* = 300 K, results in typical torsional forces ≈ 2.07 pN for *TQ* = *k*_B_ T. The excess energy pumped into the system by the means of activity is dissipated through the Langevin thermostat as detailed later. We note that in the limit of diminishing activity β*TQ* ≈ 0, the polymer behaves as a *nicked* DNA ring, which can fully relax the torsional stress within. For the high values of β*TQ*, torque applied to the two monomers excites the elastic response of the sequence of the dihedral potentials along the contour, propagating excess torsional stress over the polymer, until reaching a steady-state as explained in detail in the discussion of the results. In summary, the supercoiling agent (e.g., gyrase) increases the absolute value of supercoiling density contained within the ring, with higher value of β*TQ* resulting in a higher absolute value of supercoiling.

### IV.III Simulation Method

To sample the configurations we propagate the particles using the LAMMPS (29Oct2020 version)^68,84^ implementation of molecular dynamics with Langevin thermostat with friction γ. Translational degrees of freedom are propagated using the equation of motion

10 $$m\ddot{r_{i}}\left(t\right)=F_{i}-m\gamma {ṙ}_{i}\left(t\right)+Y_{i}\left(t\right)$$

where *m* is particle mass, **F**_*i*_ is the force on particle *i* originating from the potentials and active forces, **Y**_*i*_(*t*) is a random force obeying fluctuation–dissipation theorem such that ⟨**Y**_*i*_(*t*)⟩ = 0 and $\langle Y_{ia}(t)Y_{jb}(t^{\prime })\rangle =2\gamma mk_{B}T\delta_{ij}\delta_{ab}\delta (t-t^{\prime }),$ where δ is the Kronecker delta and indices *a*,*b* label the cartesian coordinates. Simultaneously, rotational degrees of freedom are propagated under the action of the active torques and torques from the angular potentials. We use γ = 1.0/τ, where τ = (*m*σ^2^/*k*_B_ T)^−1/2^ = 1/γ = 1 = 1000Δ*t* is the time unit and Δ*t* = 0.001τ is the integration time step.

To prepare the initial configurations for the nonequilibrium simulations, we first simulate a solution of flat ribbons (σ = 0) in equilibrium. We initialize the ring polymers as circles, placed on a well spaced primitive cubic lattice, making sure that the rings are unknotted and unlinked. We then iteratively thermalize the system, and compress it by rescaling the coordinates by a factor (*L*_0_ – *s*)/*L*_0_, where *L*_0_ is the box length before the rescaling. We repeat the sequence of the two steps until reaching the desired concentration of ρ *s* ^3^ = 0.08, in our case over a cumulative simulation length of 10^6^ τ, exceeding the Rouse time τ_RS_ ∼ (400)^2^ ∼ 10^5^ τ. Upon reaching the final density, we equilibrate the system for another 4 × 10^6^ τ and then collect the equilibrium properties over the course of final time interval 8 × 10^6^ τ. Each ring traverses a distance of several of its radii of gyration during the simulation. For the nonequilibrium simulations, we start from an equilibrium snapshot, apply the active torques and then run the simulation for 4 × 10^6^τ. For each value of torque, we simulated four independent replicas with varying initial configurations and different random seeds, and the presented ensemble averages of total mean square displacements are averaged over all four runs.

### IV.IV Threading and Branching Analysis

To detect the threadings we use the same method as detailed in ref 85. In short, at every analyzed snapshot a disk-like surface, triangulated to 4*N* triangles, is spanned on every ring and the surfaces are independently minimized by moving of the triangle vertices that do not belong to the boundary (the ring). In rare cases where the minimization does not converge,^85^ we do not analyze the threading of that snapshot. Subsequently, we detect the intersections of the contour of one ring with another ring’s minimal surface, signifying a threading of the latter by the former. The minimal surface of a threaded ring splits the threading ring in consecutive segments of lengths *L*_1_, *L*_2_, ··· *L*_*p*_, where *p* is the number of piercings through the surface. As the rings are nonconcatenated *p* is an even number and the subsequent segments are located at the opposite sides of the minimal surface.^85^ We quantify the depth of the threading by the so-called separation length *L*_sep_ defined by

11 $$L_{\mathrm{sep}}=\min\left(\sum_{i\;\mathrm{even}}L_{i},\sum_{i\;\mathrm{odd}}L_{i}\right)$$

The separation length *L*_sep_ ∈ [0, *N*/2] quantifies the amount of the polymer material of the threading ring on one of the two sides of the minimal surface of the threaded ring.

To describe the threading network, for each configuration, we construct a connectivity matrix A of size *M* × *M*, such that if ring 0 ≤ *i* < *M* threads ring 0 ≤ *j* < *M* with *L*_sep_ > *L*_sep_^thr^, then A[i,j]=1 , otherwise A[i,j]=0 . This matrix in turn defines a graph of *M* nodes, where a threading event corresponds to a directed edge. Within this graph, we localize the maximal connected components corresponding to threaded clusters. We consider *L*_sep_^thr^ = 0 unless stated otherwise.

To estimate the number of branches, we detect the tips of plectonemes by calculating the profile of local segmental writhe, *W*(*i*),^48,86^ for each monomer *i* as

12 $$W\left(i\right)=\frac{1}{4\pi }\int_{C_{1}}\int_{C_{2}}\frac{r_{1}-r_{2}}{|r_{1}-r_{2}{|}^{3}}\left({ṙ}_{1}\times {ṙ}_{2}\right)ds_{1}ds_{2}$$

where **r**_*i*_ = **r**_*i*_(*s*_*i*_) is a parametrization of curve *C*_*i*_ with arc length *s*_*i*_ and curves *C*_1_: **r**_1_(*s*_1_) and *C*_2_: **r**_2_(*s*_2_) follow the polymer contour between the monomers [*i* – *w*, *i*] and [*i*, *i* + *w*] respectively, where *w* = 20 covers the segment length of approximately one persistence length. The local maxima of *W*(*i*) correspond to the tips of branches, and we also require that *W*(*i*) ≥ *W*_thr_ at these maxima, where *W*_thr_ is the threshold set to avoid the false branch recognition, representing only topologically insignificant local segmental bending due to fluctuations. Number of branches *n*_br_ on a polymer is then defined as the number of local maxima on the segmental writhe profile. In essence the threshold defines what is considered a branch. Branch configuration with a locally open (flat nonsupercoiled) tip, for example due to a threading, might exhibit smaller value of *W* and hence can lead to undercounting of the number of branches. In contrast, a long branch, if wrapped around itself, can locally exhibit higher *W* even at segments that are not at its tip, that can lead to overcounting of the number of branches. The latter effect can be reduced by the use of relatively short segment length *w*, as we do, that is relatively stiff and reduces such bending. We show the mean number of branches and the standard deviation of their distribution as a function of the threshold in Figure S4 showing that our two conclusions from the Results section, namely (i) branching being the increasing function of the torque at time *t* = 10^5^τ and (ii) the branching is strongly reduced to the extent of linear-like chains at *t* = 10^6^τ, hold irrespective of the specific value of the threshold (*W*_thr_ ≥ 0.2). We confirmed visually in a number of cases that the branching is correctly captured. To produce the figure, Figure 4c we used the threshold *W*_thr_ = 0.35 as in ref 48.

### IV.V Effective Monte Carlo Simulations

The effective 1D Monte Carlo simulation based on the work of Lo and Turner^75^ treats each ring as a linear doubly folded chain, of effective length *N*_eff_, that can be passively threaded or can actively thread other chains at some contour position *x*_*a*/*p*_ ∈ [0, *N*_eff_ – 1]. At every step each chain is selected in random order and a random move of unit length left/right is attempted. If a passive threading exists at a contour coordinate *x*_*p*_ = 0 or *x*_*p*_ = *N*_eff_ – 1 the left/right move is rejected, respectively. If move is accepted the chain is shifted, we update the position of the chain, the locations of all the passive threadings *x*_*p*_ of the given chain and the locations of all the active threadings *x*_*a*_. If an active threading location *x*_*a*_ moves out of bounds [0, *N*_eff_ – 1] the threading is relaxed and vanishes. The initial threading network for the effective simulation is from our MD simulations at time *t* = 10^5^τ. We use the information on ring pairs that are in threading configuration, which of them is passively/actively threaded and we use the value of *L*_sep_ as a proxy for the *x*_*a*_. Specifically, *x*_*a*_ = *L*_sep_ *N*_eff_/(*N*/2). Unfortunately from the threading analysis Section 4.4 we do not have access to the locations of passive threadings because these correspond to the locations of the intersections of the minimal surfaces and not of the ring contours, hence are more difficult to define. Therefore, we tested our simulations for two setups. First, the locations of the passive threadings are chosen uniformly randomly *x*_*p*_ ∈ [0, *N*_eff_ – 1]. Such a choice can however lead to stalled global conformations, in particular when there exist many mutual threadings and the effective ring lengths are short, because many rings can have mutually (or in cycles) *x*_*p*_ = 0 and *x*_*p*_ = *N*_eff_ – 1 making such conformations impossible to relax. More freedom can be obtained by either increasing *N*_eff_ or by restricting the initial *x*_*p*_’s to be located away from the chain ends. We comment on these options in SI (Section I A).

## Supplementary Material



## Notes

Effective Monte Carlo Simulations, comparison with different microscopic model, additional simulation results: polymer shape parameters, branching analysis for threaded and dangling ring classes, separation length distributions, network topology analysis, dynamics of deadlocked rings and dynamics of rings in steady state (PDF)
