# Supplementary material for: Actively Induced Supercoiling Can Slow Down Plasmid Solutions by Trapping the Threading Entanglements
Source: ACS Nano. 2026 May 15;20(21):14998–5007. doi: 10.1021/acsnano.5c10811 (PMC13235645; doi:10.1021/acsnano.5c10811)
Supplement: Supplementary file 1 [file nn5c10811_si_001.pdf]

Supplementary Information: Actively Induced Supercoiling Can Slow Down Plasmid Solutions by Trapping the Threading Entanglements

Roman Staňo,[1,2] Renáta Rusková,[3,4] Dušan Račko,[3,*] and Jan Smrek[1,†]

[1]Faculty of Physics, University of Vienna, Boltzmanngasse 5, 1090 Vienna, Austria
[2]Yusuf Hamied Department of Chemistry, University of Cambridge, Lensfield Road, Cambridge CB2 1EW, UK
[3]Polymer Institute, Slovak Academy of Sciences, Dúbravská cesta 9, 845 41 Bratislava, Slovakia
[4]Department of Physics, Sapienza University of Rome, Piazzale Aldo Moro 5, 00185 Rome, Italy
(Dated: May 15, 2026)

CONTENTS

I. Models & Methods 1
A. Effective Monte Carlo Simulations 1
B. Original Reference Model 1

II. Additional Simulation Results 4
A. Shape Parameters 4
B. Branching Analysis 5
C. Separation Length Distributions 7
D. Network Topology Analysis 9
E. Dynamics of Threaded Rings 10

References 12

I. MODELS & METHODS

A. Effective Monte Carlo Simulations

In the effective 1D Monte Carlo simulation we track the mean-squared displacement of the chains and the system relaxation time defined as the time until the last threaded ring pair unthreads. In Fig. S1 we show the results for systems with $N_{\text{eff}} = 20$ and initial $x_p = N_{\text{eff}}/2$ for all the threadings. The mean-squared displacement shows a prolonged subdiffusion and longer relaxation times for systems with smaller active torques that exhibit relatively more threadings per ring. These observations are consistent with equilibrium simulations [1] that show significant increase of relaxation time with the number of threadings. Another set of simulations with uniformly distributed x_p typically stall for lower torques, as explained in the main text, as a fraction of the rings remain trapped in a threading cluster. The fraction is higher for lower torques consistent with a higher number of threadings. In the last set of simulations we used $N_{\text{eff}} = 100$ and uniformly distributed x_p, leading to qualitatively similar results to the ones in Fig. S1 (not shown) but poor statistics due to long simulation times.

B. Original Reference Model

Herein, we compare our current polymer model (Sec. IV.I) with the original one, which was used in earlier studies by some of us [2] and designed to quantitatively reproduce the topological aspects of DNA [3–5]. The implementation of the model we have at hand (in a non-maintained version ESPResSo-3.1 [6, 7]) appears slower than the implementation of the model in Sec. IV.I, presumably because of the larger number of particles and older software of the former, but no direct detailed comparison of the performance was done. Although the current model [8] appears more efficient, it was not tuned to quantitatively emulate all of the properties of DNA. The point of comparison was to demonstrate, that the observed phenomena are robust to the used model and they agree with the previously published results.

* dusan.racko@savba.sk
† jan.smrek@univie.ac.at

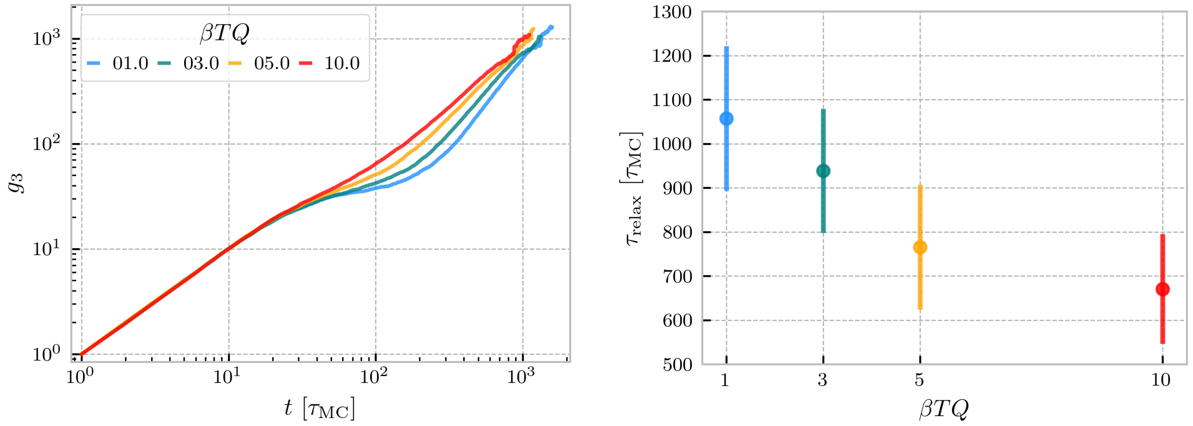

FIG. S1. Mean-squared displacement g_3 (left) and relaxation time (right) $\tau_{\rm relax}$ of rings from the effective MC simulations as specified in Sec. I A. The presented data are the average of 50 samples where each sample had the same initial configuration specified in the text, but likely different sequence of random numbers. The error-bars of $\tau_{\rm relax}$ represent the standard deviation of the sample. $\tau_{\rm MC}$ is the Monte Carlo time (number of steps) and g_3 is dimensionless as it refers to the MSD in the space of particle indices.

In the original model, a monomeric unit, i, is composed of a single bead with $s = 2.5$ nm, and five virtual sites, labeled as $p_{1i}, p_{2i}, p_{3i}, p_{4i}, a_i$. All of the virtual sites lie on the same plane, having positions $\{p_{1i} : [-x, 0]; p_{2i} : [x, 0]; p_{3i} : [0, -x]; p_{4i} : [0, x]; a_i : [0, 0]\}$ and this plane is placed perpendicularly to the bond vector connecting bead i and $i + 1$, where virtual beads form a cross with the arm length $x = 0.9s$ (see below the potential details). Beads of consecutive monomeric units are bonded with a harmonic bond

$$U_{\rm h}(r) = \frac{1}{2} K_{\rm h}(r - r_0)^2, \tag{S1}$$

with $K_{\rm h} = 100k_{\rm B}T/s^2$ and $r_0 = s$. The non-bonded interactions between beads of any two monomeric units are modeled using WCA with the form Eq. (3) with $\varepsilon = k_{\rm B}T$, while virtual sites have no non-bonded interactions. The polymer bending is emulated with harmonic potential:

$$U_{\rm b}(r) = \frac{1}{2} K_{\rm b}(\theta - \theta_0)^2, \tag{S2}$$

with $K_{\rm b} = 20k_{\rm B}T$ and $\theta_0 = \pi$, and θ being the angle between two consequent bond vectors, altogether yielding the persistence length of $l_{\rm p} \approx 50$ nm. To model the torsion, we utilize harmonic dihedral potentials

$$U_{\rm d}(r) = \frac{1}{2} K_{\rm d}(\phi - \phi_0)^2, \tag{S3}$$

with $K_{\rm d} = 25k_{\rm B}T$, $\phi_0 = 0$ and ϕ being the angle between two planes – one defined by the triplet of particles $\{a_i, a_{i+1}, p_{1i}\}$, the other by the triplet $\{a_i, a_{i+1}, p_{1i+1}\}$ (see also the model scheme in [2]). The choice of $K_{\rm d}$ should result in the mean excess writhe-to-twist ratio of $\approx 7 : 3$ [9]. Finally, the inner geometry of the virtual sites is fixed by a series of harmonic potentials. First a bonding potential fixing the arm length of the cross, having the stiffness $80k_{\rm B}T$ with the potential well minimum at $0.9s$ applied between pairs $(a_i, p_{1i}), (a_i, p_{2i}), (a_i, p_{3i}), (a_i, p_{4i})$. Second a bonding potential fixing the position of the virtual sites relative the monomeric units, having the stiffness $80k_{\rm B}T$ with the potential well minimum at $s/2$ applied between pairs $(i, a_i), (i + 1, a_i)$. Third, a bending angular potential controlling the relative orientation of the neighboring virtual sites, having the stiffness $50k_{\rm B}T$ with the potential well minimum at $\pi/2$ applied at triplets $(p_{1i}, a_i, p_{3i},), (p_{1i}, a_i, p_{4i},), (p_{2i}, a_i, p_{3i},), (p_{2i}, a_i, p_{4i})$. Finally, a bending angular potential for keeping the virtual sites in the single plane, having the stiffness $50k_{\rm B}T$ with the potential well minimum at π applied at triplets $(p_{1i}, a_i, p_{2i}), (p_{3i}, a_i, p_{4i})$.

To simulate the effect of the supercoiling agent, we remove the dihedral potential from a pair of consecutive monomeric units, or rather their virtual sites, and instead apply a constant torque TQ in the form of constant force F acting on the cross arms, rotating them around the axis of the bond

We used systems with $M = 50$ rings of length $N = 400$ ($\approx$ 3kbp) in a cubic box with periodic boundary conditions at monomer density $\rho\sigma^3 = 0.08$. We use the ESPResSo-3.1 implementation of Langevin dynamics as formulated in

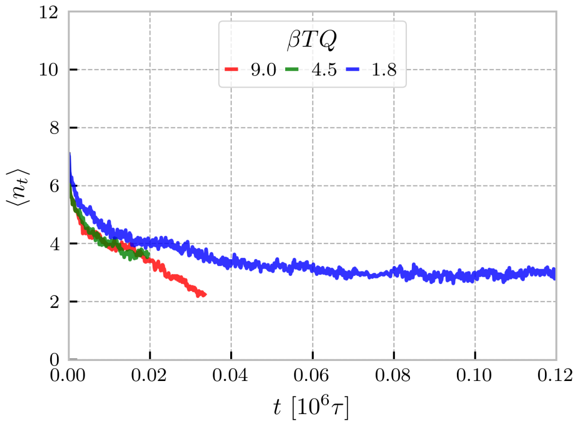

FIG. S2. Time series of the number of threadings per ring for different torques for the original model from Section I B. We use $\beta TQ = Fl$, where F is the input force of the model and $l = 0.9s$ is the arm length, as described in Section I B.

Eq. (10), but with $\Delta t = 0.0012\tau$ and $\gamma\tau = 3.9$, and with total length of $\sim 10^5\tau$ just slightly above the Rouse time of a single ring.

In Fig. S2 we show the number of threadings in time for the original model. The comparison with the Fig. 3 in the main text shows that the observed phenomena (two regimes of the n_t decay) are universal and robust to the choice of model. The small quantitative disparities (somewhat smaller n_t) are caused by the smaller system size, where a ring could in principle interact and entangle with itself through the boundary conditions. Such events decrease the measured n_t because we do not detect self-threadings. Further differences between the current and the original model constitute the interaction potentials, namely different effective torsional stiffness and tilting potential combined with presence of additional degrees of freedom in the original model – patchy monomeric unit vs. rigid bodies with inscribed directionality. Note that the longest simulations in Fig. S2 are only of the order of the Rouse time of the rings. Although longer simulations are possible in principle, we have not used the original model for production simulations.

II. ADDITIONAL SIMULATION RESULTS

A. Shape Parameters

To describe the size and shape of the rings, we use the radius of gyration, R_{g} and relative shape anisotropy κ^2 respectively. For each of the rings in a given time, we first construct the gyration tensor, $\mathcal{G}$ with 3×3 components

$$\mathcal{G}_{xy} = \frac{1}{2N^2} \sum_{i=1}^{N} \sum_{j=1}^{N} (x_i - x_j)(y_i - y_j), \tag{S4}$$

where x_i (or x_j) is the cartesian x coordinate of the monomeric unit i (or j), analogously defined for axes $[x, y, z]$. Diagonalization of this gyration tensor yields three eigenvalues $\lambda_1^2 \leq \lambda_2^2 \leq \lambda_3^2$ such that $\mathrm{Tr}(G) = \lambda_1^2 + \lambda_2^2 + \lambda_3^2 = R_{\mathrm{g}}^2$. We define the relative shape anisotropy [10] as

$$\kappa^2 = \frac{3}{2} \frac{\lambda_1^4 + \lambda_2^4 + \lambda_3^4}{(\lambda_1^2 + \lambda_2^2 + \lambda_3^2)^2} - \frac{1}{2}, \tag{S5}$$

which takes values between zero and one, with zero being attained only for spherically symmetric objects and one occurring only for linear objects. In Fig. S3 we then present expectation values $\langle \cdot \rangle$ of these single-ring properties, obtained by averaging over all of the rings in the system.

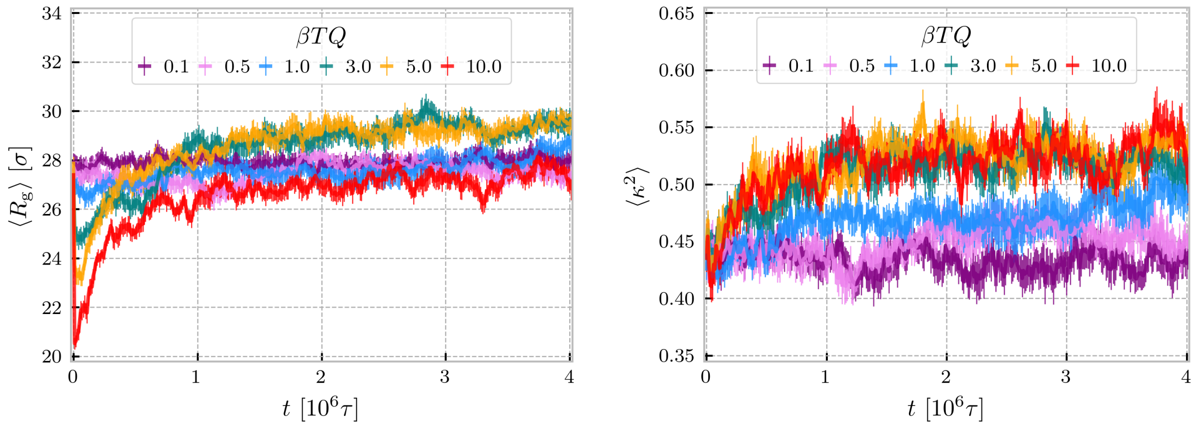

FIG. S3. Time series of the mean radius of gyration $\langle R_{\mathrm{g}} \rangle$ and the mean shape anisotropy $\langle \kappa^2 \rangle$ averaged over all rings, plotted for different values of the active torque. The error bars are the error of the mean.

B. Branching Analysis

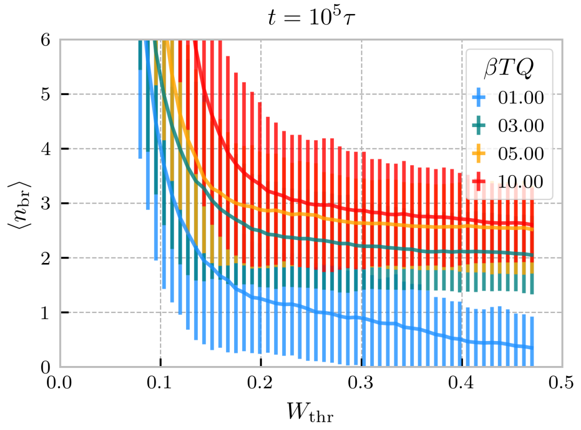

FIG. S4. Mean number of branches as a function of the threshold value of segmental writhe as detailed in Sec. IV.IV, plotted for different torques at two different times – at the end of the initial stage of supercoiling and during the slow aging regime respectively. The error bars are the standard deviation over the ensemble of rings.

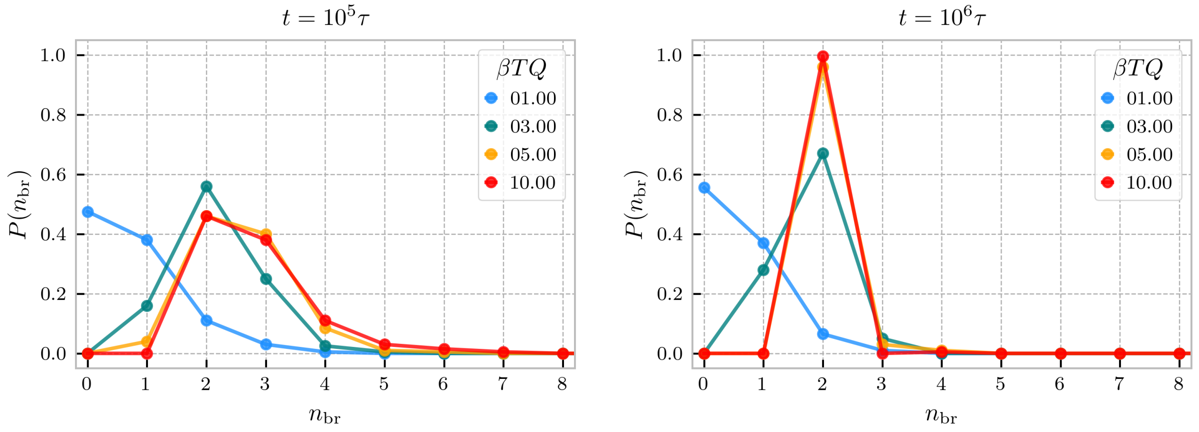

FIG. S5. Normalized histograms of the number of branches for different torques at two different times – at the end of the initial stage of supercoiling tightening and during the slow aging regime respectively.

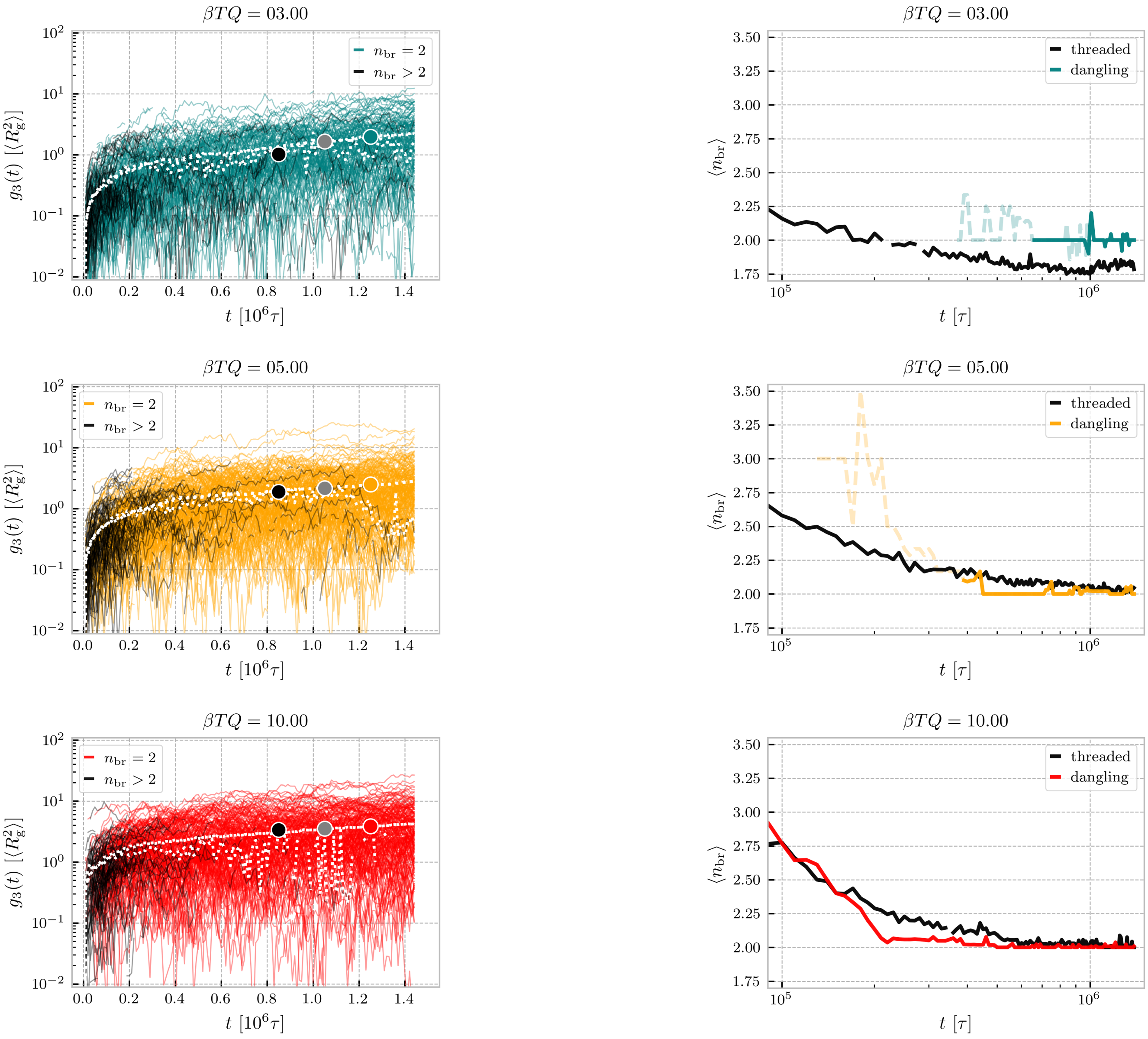

FIG. S6. Left: The mean square displacement of all individual rings, with the point at time t colored black if the ring has more than two branches. Three white dotted lines show means over three subpopulations of rings – over the rings with two branches, more than two branches and over all of the rings. Right: The mean branching averaged over two populations of rings – threaded and dangling. Partially translucent dashed lines signify that less than ten rings contribute to the mean causing large fluctuations, solid full bodied line is considered to be statistically more significant. The few breaks in black solid lines are snapshots that were not analyzed due to threading detection method (see Sec. IV.IV).

C. Separation Length Distributions

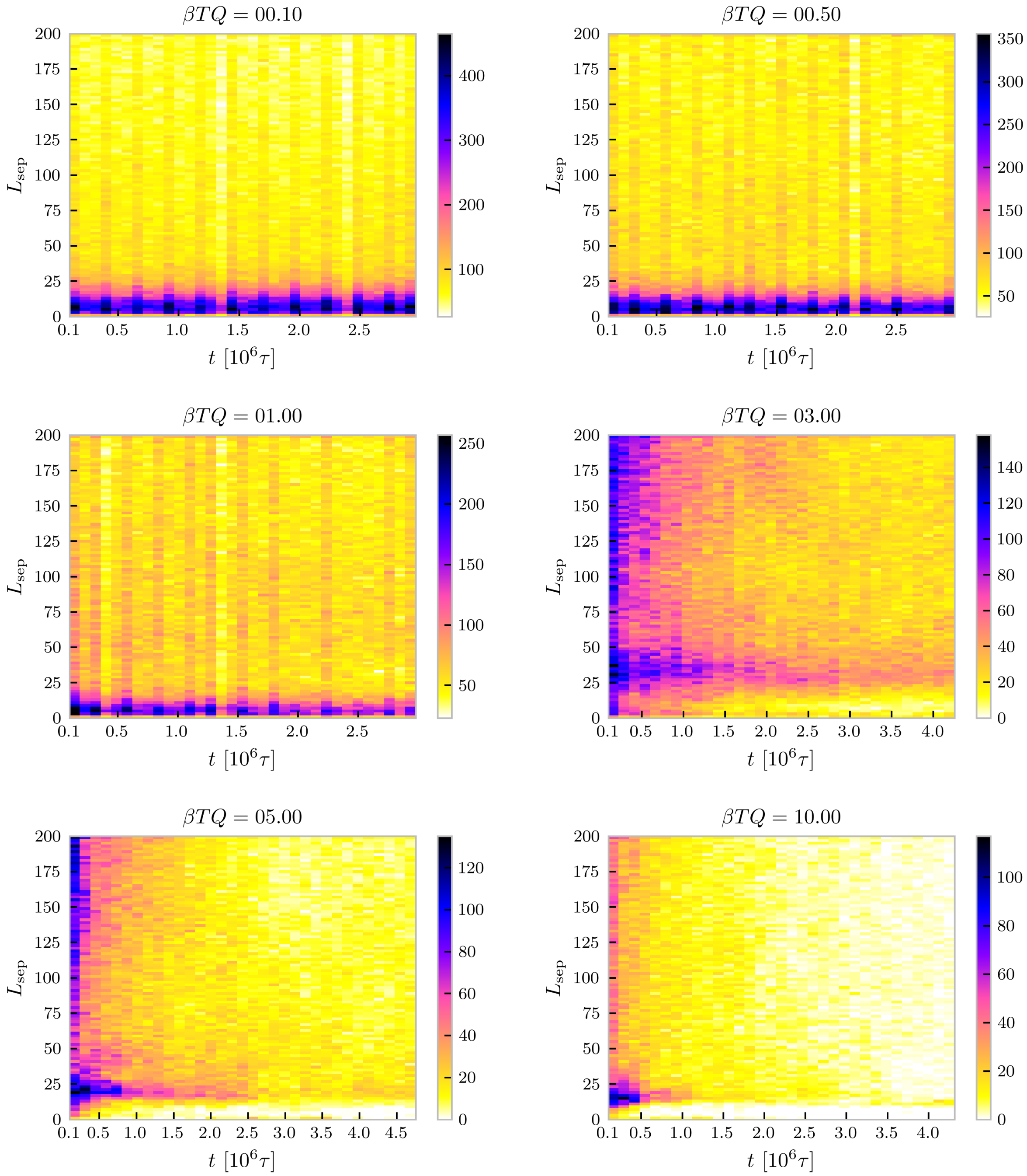

FIG. S7. Two dimensional probability distributions of separation length, $L_{\rm sep}$, Eq. (11), in time. The color code corresponds to the number of threadings of a given separation length in a given time. The time axis starts at $10^5\tau$, only after the initial stage of rapid supercoiling.

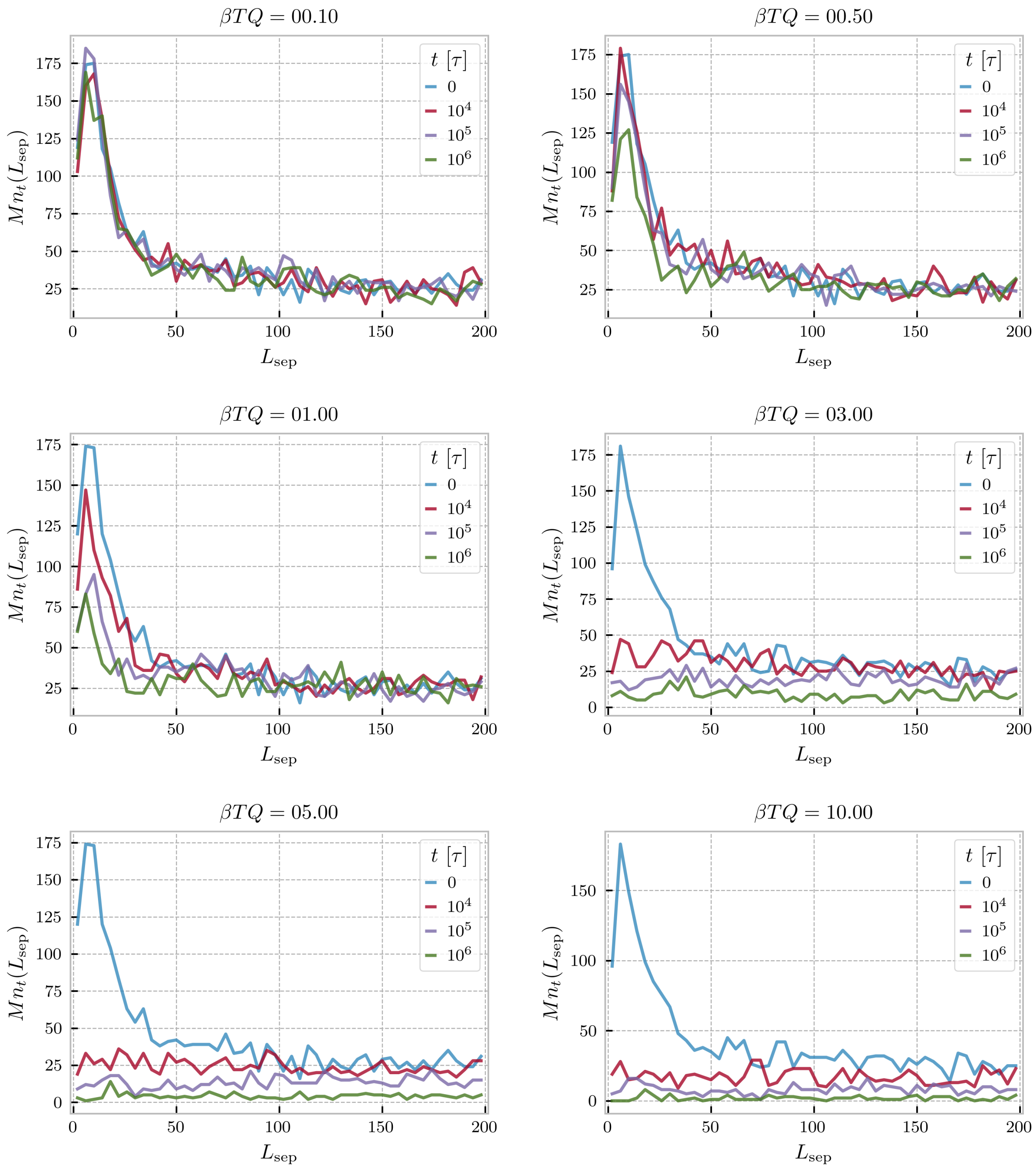

FIG. S8. Total number of threadings of separation length L_{sep} observed in the system of $M = 200$ rings plotted for three different times – during the stage of rapid supercoiling; during the transition from rapid supercoiling to the slow tightening regime; during the regime of slow aging – in the order of increasing time. Time $t = 0$ corresponds to the initial (equilibrium) configuration. Presented histograms are essentially vertical slices of the Fig. S7.

D. Network Topology Analysis

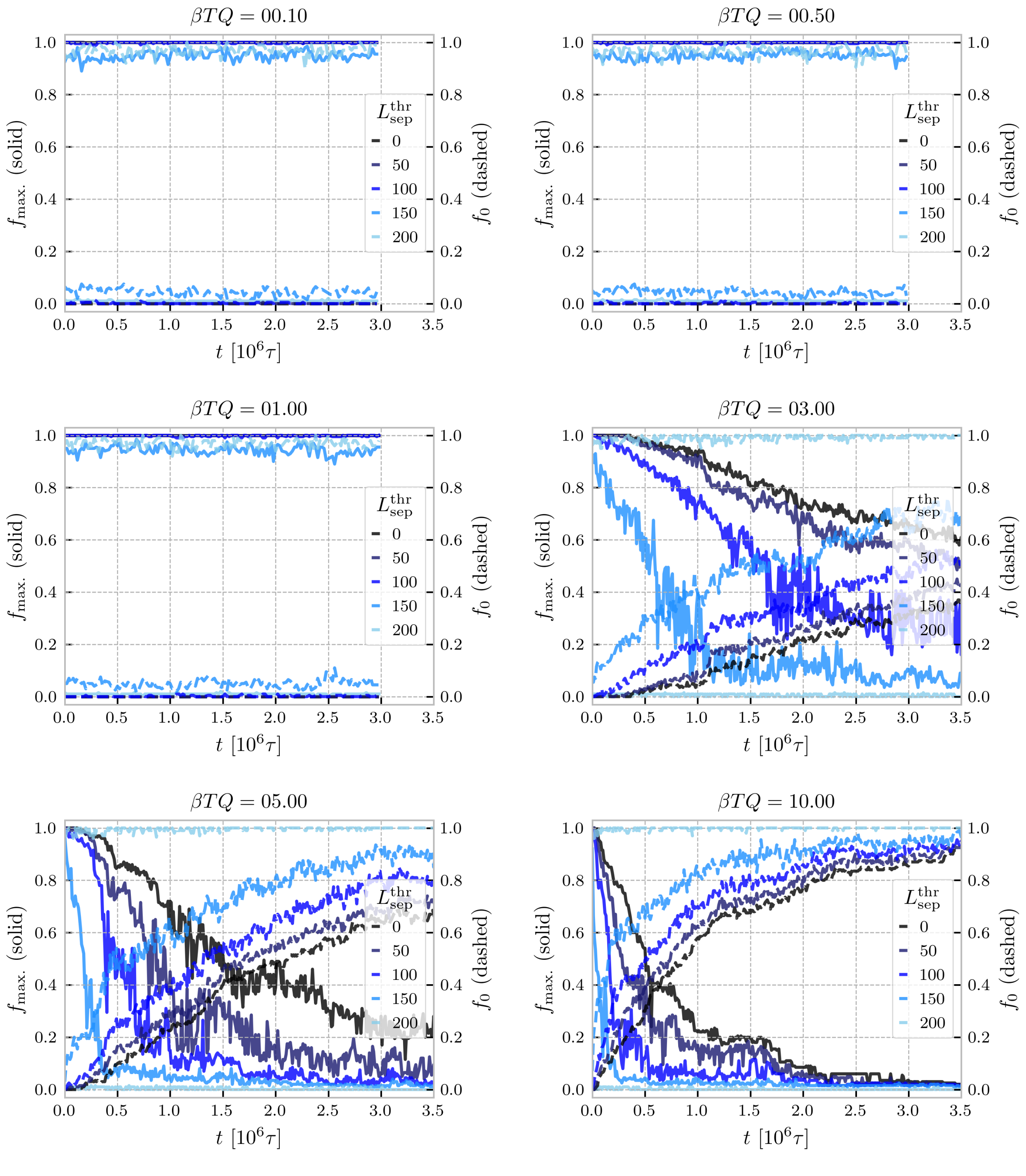

FIG. S9. Time series of network properties for different torques and different threshold values $L_{\mathrm{sep}}^{\mathrm{thr}}$ used in the definition of the network following the Sec. IV.IV.

E. Dynamics of Threaded Rings

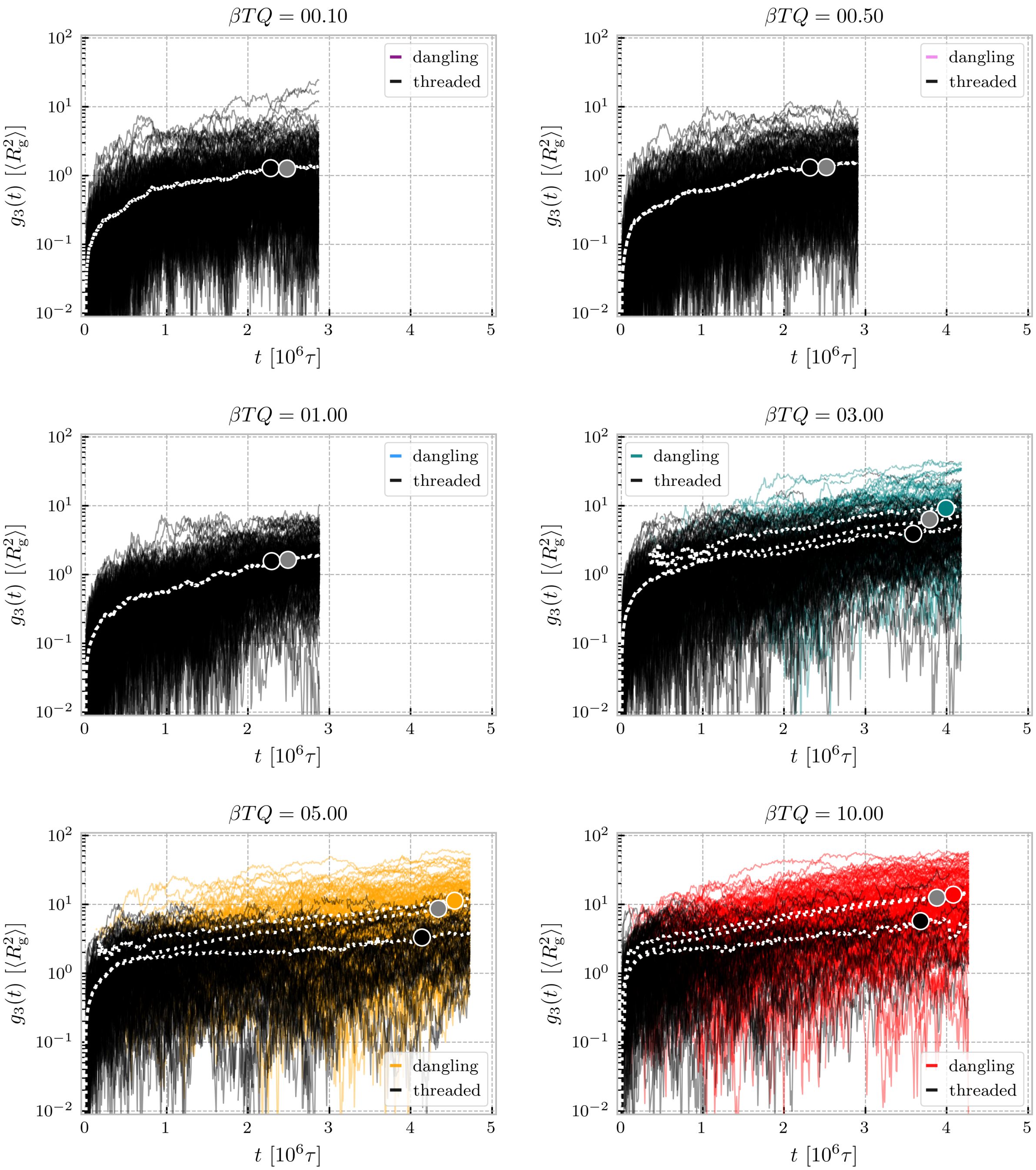

FIG. S10. The mean square displacement of all individual rings, with the point at time t colored black if the ring is threaded or colored if it is dangling, plotted for different torques. White dotted lines show means over three subpopulations of rings – over all dangling rings, over all threaded rings and over all of the rings, marked with color, black and gray circular marker respectively. In some of the cases, we have no dangling rings.

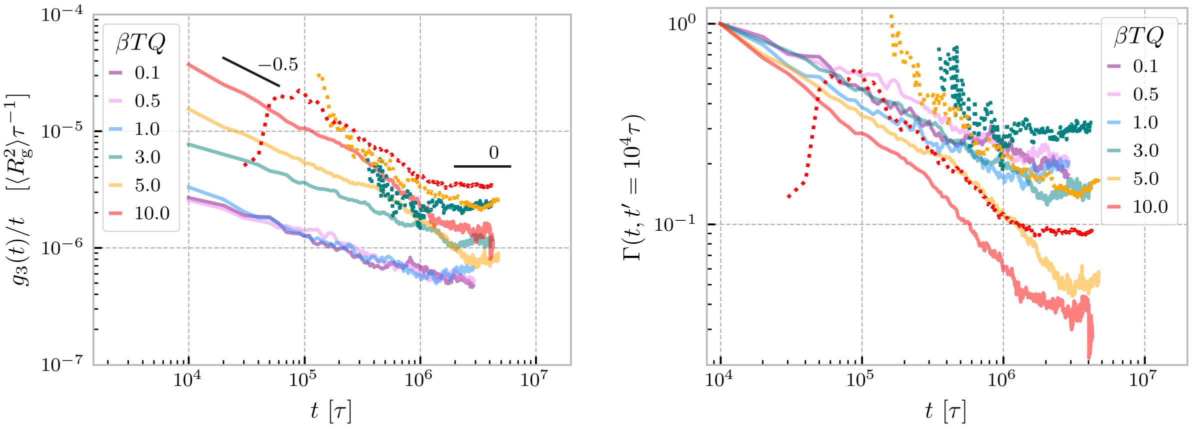

FIG. S11. The mean square displacement divided by time (left) and relaxation functions (right) plotted for different torques. Solid lines denote the averages over the threaded rings only, while the dotted lines are averaged over the dangling rings only. Note that the dangling rings emerge only for the three largest values of βTQ. Since dangling rings emerge only in the long time limit, the normalization factor of the relaxation function $\Gamma(t, t')$ is strictly defined only for the threaded rings. To plot the functions for the dangling rings as well, we just use the normalization of the threaded ones at the same TQ, hence the dotted lines in $\Gamma(t, t')$ do not start at one.

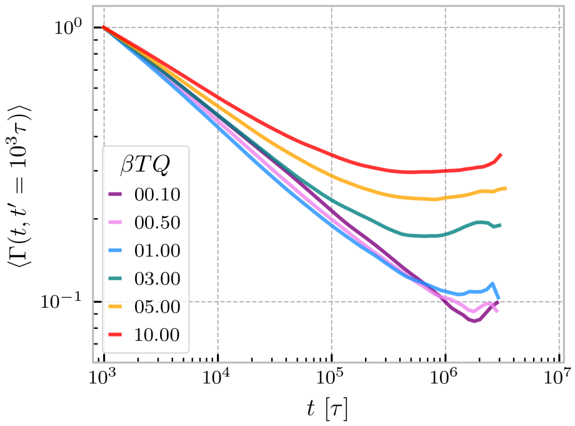

FIG. S12. The averaged relaxation function Γ plotted for different torques in the limit of long times. In this case, we redefine the time zero in the corresponding $g_3(t)$ as $t_0 = 10^6\tau$ as opposed to $t_0 = 0$ in all other plots of Γ. This change means that the function presented here probes only the relaxation in the regime where the supercoiling saturates (Fig. 1d), long after the initial rapid supercoiling period. For every lag time t, we present $\langle \Gamma \rangle$ which is Γ averaged over all possible origins after $t_0 = 10^6\tau$. This relaxation function is hence equivalent to equilibrium relaxation of rings with the supercoiling degree corresponding to its saturated value at the given βTQ. It shows that in equilibrium the rings with the highest supercoiling relax the fastest as seen in [11].

[1] W.-C. Lo and M. S. Turner, The Topological Glass In Ring Polymers, Europhys. Lett. **102**, 58005 (2013).

[2] D. Racko, F. Benedetti, J. Dorier, Y. Burnier, and A. Stasiak, Molecular Dynamics Simulation of Supercoiled, Knotted, and Catenated DNA Molecules, Including Modeling of Action of DNA Gyrase., Methods Mol. Biol. **1624**, 339 (2017).

[3] F. Benedetti, D. Racko, J. Dorier, and A. Stasiak, Introducing Supercoiling into Models of Chromosome Structure, in *Modeling The 3D Conformation of Genomes* (CRC Press, 2019) pp. 115–138.

[4] D. Racko, F. Benedetti, J. Dorier, and A. Stasiak, Are TADs supercoiled?, Nucleic Acids Res. **47**, 521 (2018).

[5] R. Rusková and D. Račko, Entropic competition between supercoiled and torsionally relaxed chromatin fibers drives loop extrusion through pseudo-topologically bound cohesin, Biology **10**, 10.3390/biology10020130 (2021).

[6] A. Arnold, O. Lenz, S. Kesselheim, R. Weeber, F. Fahrenberger, D. Roehm, P. Košovan, and C. Holm, Espresso 3.1: Molecular Dynamics Software for Coarse-Grained Models, in *Meshfree Methods for Partial Differential Equations VI*, edited by M. Griebel and M. A. Schweitzer (Springer Berlin Heidelberg, 2013) pp. 1–23, place: Berlin, Heidelberg.

[7] H. Limbach, A. Arnold, B. Mann, and C. Holm, Espresso—an extensible simulation package for research on soft matter systems, Comput. Phys. Commun. **174**, 704 (2006).

[8] C. A. Brackley, A. N. Morozov, and D. Marenduzzo, Models For Twistable Elastic Polymers In Brownian Dynamics, And Their Implementation For Lammps, J. Chem. Phys. **140**, 135103 (2014).

[9] J. Bednar, P. Furrer, A. Stasiak, J. Dubochet, E. H. Egelman, and A. D. Bates, The Twist, Writhe And Overall Shape Of Supercoiled DNA Change During Counterion-Induced Transition From A Loosely To A Tightly Interwound Superhelix, J. Mol. Biol. **235**, 825–847 (1994).

[10] A. Narros, A. J. Moreno, and C. N. Likos, Effects Of Knots On Ring Polymers In Solvents Of Varying Quality, Macromolecules **46**, 3654 (2013).

[11] J. Smrek, J. Garamella, R. Robertson-Anderson, and D. Michieletto, Topological Tuning Of DNA Mobility In Entangled Solutions Of Supercoiled Plasmids, Sci. Adv. **7**, eabf9260 (2021).
